# Development of a data collection and management system in West Africa: challenges and sustainability

**DOI:** 10.1186/s40249-018-0494-4

**Published:** 2018-11-16

**Authors:** Jeffrey G. Shaffer, Seydou O. Doumbia, Daouda Ndiaye, Ayouba Diarra, Jules F. Gomis, Davis Nwakanma, Ismaela Abubakar, Abdullahi Ahmad, Muna Affara, Mary Lukowski, Clarissa Valim, James C. Welty, Frances J. Mather, Joseph Keating, Donald J. Krogstad

**Affiliations:** 1 Departments of Biostatistics (1440 Canal St., Suite 1610) and Tropical Medicine, (#8317 1430 Tulane Avenue, J.B. Johnston Building, Room 510), New Orleans, LA 70112-2699 USA; 2 University of the Sciences, Techniques and Technologies of Bamako, Bamako, Mali; 3 University Cheikh Anta Diop, Dakar, Senegal; 4 Medical Research Council Unit, Fajara, The Gambia; 5 ScienceTRAX LLC, Macon, GA USA; 6 Harvard T.H. Chan School of Public Health, Boston, MA USA

**Keywords:** Data collection, Data (database) management system, Diseases of poverty, Malaria, *Plasmodium falciparum*

## Abstract

**Background:**

Developing and sustaining a data collection and management system (DCMS) is difficult in malaria-endemic countries because of limitations in internet bandwidth, computer resources and numbers of trained personnel. The premise of this paper is that development of a DCMS in West Africa was a critically important outcome of the West African International Centers of Excellence for Malaria Research. The purposes of this paper are to make that information available to other investigators and to encourage the linkage of DCMSs to international research and Ministry of Health data systems and repositories.

**Methods:**

We designed and implemented a DCMS to link study sites in Mali, Senegal and The Gambia. This system was based on case report forms for epidemiologic, entomologic, clinical and laboratory aspects of plasmodial infection and malarial disease for a longitudinal cohort study and included on-site training for Principal Investigators and Data Managers. Based on this experience, we propose guidelines for the design and sustainability of DCMSs in environments with limited resources and personnel.

**Results:**

From 2012 to 2017, we performed biannual thick smear surveys for plasmodial infection, mosquito collections for anopheline biting rates and sporozoite rates and year-round passive case detection for malarial disease in four longitudinal cohorts with 7708 individuals and 918 households in Senegal, The Gambia and Mali. Major challenges included the development of uniform definitions and reporting, assessment of data entry error rates, unstable and limited internet access and software and technology maintenance. Strengths included entomologic collections linked to longitudinal cohort studies, on-site data centres and a cloud-based data repository.

**Conclusions:**

At a time when research on diseases of poverty in low and middle-income countries is a global priority, the resources available to ensure accurate data collection and the electronic availability of those data remain severely limited. Based on our experience, we suggest the development of a regional DCMS. This approach is more economical than separate data centres and has the potential to improve data quality by encouraging shared case definitions, data validation strategies and analytic approaches including the molecular analysis of treatment successes and failures.

**Electronic supplementary material:**

The online version of this article (10.1186/s40249-018-0494-4) contains supplementary material, which is available to authorized users.

## Multilingual abstracts

Please see Additional file 1 for translations of the abstract into five official working languages of the United Nations.

## Background

Malaria is a disease of poverty [1–3]. Although many government programs focus on malaria prevention and treatment, children and adults living in poverty have increased risks of plasmodial infection and malarial disease. In addition, those risks may be exacerbated by limited access to long-lasting insecticidal nets (LLINs) and Intermittent Preventive Treatment during pregnancy (IPTp) and by the cost of treatment with Artemisinin Combination Therapies (ACTs), especially when supplies are low or absent during stockouts. Likewise, poverty limits the resources available for computers with greater processing speed and storage capacity and the training of host-country investigators and thus the ability of malaria-endemic countries to identify their most frequent health problems and compare the effectiveness of different control strategies. Because of those concerns, we propose the development of data collection and management systems (DCMSs) in areas such as West Africa to increase the availability of quality epidemiologic data which are often unavailable or delayed and to support the analysis of data from population- and health facility-based studies. Although a Health Management Information System provides greater opportunity for the improvement of health than a DCMS, it also requires a greater investment at a time when the resources available in West Africa are severely limited. For that reason, in this paper we have focused on development of a DCMS which was feasible with the resources available through an International Centers of Excellence for Malaria Research (ICEMR) grant award provided by National Institutes of Allergy and Infectious Diseases.

**Fig. 1:**
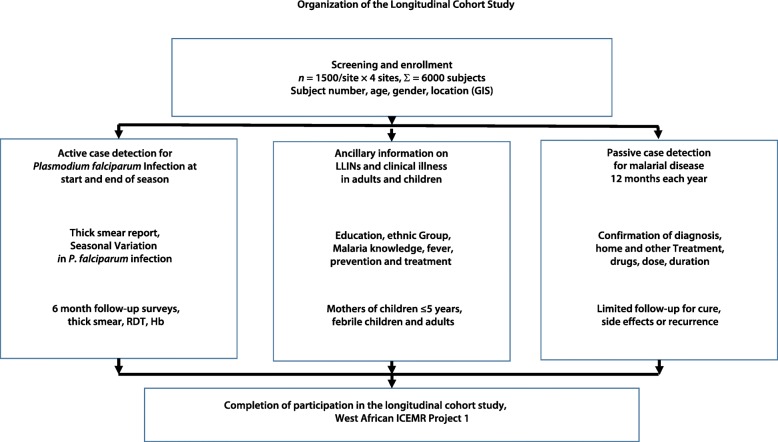
Organization of the Longitudinal Cohort Study. With four study sites in three countries, this longitudinal study examined the prevalence of *P. falciparum* infection by Active Case Detection (biannual thick smears, ACD) and the incidence of disease by Passive Case Detection (PCD). Information from household surveys and data from ACD and PCD were recorded on Case Report Forms (CRFs) and entered in a computerized database using the *StudyTRAX* software. GIS: Geographic information system; ICEMR: International Center of Excellence for Malaria Research; LLIN: Long-lasting insecticidal net; RDT: Rapid diagnostic test

With increased health research in low and middle-income countries (LMICs) during the past decade, the role of data systems in improving health outcomes is receiving greater attention. For example, recent reports indicate that a number of countries fall short on metrics such as health records and policy planning [4–7]. Frequent problems include insufficient procedures for the collection, storage, analysis and distribution of data and insufficient numbers and capacity of servers and data repositories [8]. Because of weak data systems, organizations such as the International Network for the Demographic Evaluation of Populations and Their Health (INDEPTH) network in Accra, Ghana and Pune, India rely on empiric approaches to monitor demographic and health outcomes [9, 10]. This challenge is exacerbated by the increasing dependence of modern data systems on technologies and expertise that are less available in Africa. For example, additional training in data interpretation and more flexible data systems were necessary to improve health services in Tanzania [11]. According to the World Health Organization (WHO), strategies for improving data systems should begin by examining the existing systems while tracking challenges and suggestions for improvement [12]. Likewise, investigators have long argued that stronger health systems and better documentation are necessary to understand the successes and failures of health systems in developing countries [13]. The premise of this paper is that developing a DCMS for study sites in Mali, Senegal and The Gambia (Fig. 1) was a critically important outcome of the West African ICEMR [14, 15].

Here we consider the role of a DCMS from the initial steps of data collection and management to the final steps of analysis and reporting. The primary components of a DCMS include: 1) data collection, 2) data collection personnel, 3) a centralized reporting system and 4) data management personnel. DCMSs are within the larger domain of health information systems [12] and centralized reporting systems are commonly developed from relational databases. When resources and internet bandwidth permit, it is desirable to place databases on cloud-based servers to ensure single data sources and prevent the loss of data from environmental hazards and technical failures. Please note, however, that the use of cloud-based servers creates consent challenges and increases ethical concerns related to the privacy and confidentiality of the data. For those reasons, de-identified data in offline databases such as *Access* (Microsoft, Redmond, WA, USA) may be preferable at sites with limited bandwidth and internet access. Finally, multi-country or regionally-based DCMSs are appealing because they potentially benefit from access to diverse populations, provide opportunities to pool resources and data and potentially facilitate the comparison and standardization of data over time. In addition, DCMSs serving different countries need to standardize definitions to integrate and interpret data from multiple sites. Please note, that this process may be complicated by cultural/religious and policy differences, especially when working across international boundaries.

An effective DCMS requires accurate and timely data collection and reporting, adequate infrastructural and technical support, trained personnel and sufficient funding to sustain the system. However, because these requirements vary with the resources available (fewer local resources → greater need for external support), DCMS strategies vary by country. For example, in Sierra Leone, where only 20% of the population had access to electricity in 2016 [16], offline databases are used frequently [17]. Conversely, in Mali, where electricity is more available, a cloud-based DCMS has been used. However, the value of wealthier countries as comparators may be limited even within regions because differences in the Gross Domestic Product (GDP) also affect the availability of resources. For example, the GDP per person in South Africa is 10-fold higher than in other sub-Saharan countries: USD 5285 vs USD 505, USD 780, USD 473 and USD953 for South Africa vs Sierra Leone, Mali, The Gambia and Senegal [18, 19].

In terms of financial support, the short-term nature of most funding for data systems is the greatest limitation. Moucheraud et al. have recently discussed strategies for sustaining health information systems [20] and have emphasized the benefits of systems with clearly defined goals.

The ICEMR Network was established in 2010 to identify and resolve obstacles to malaria control in 20 malaria-endemic countries [21]. Gutierrez et al. have discussed the general approach to data integration across the ICEMR study sites [22]. The goal of the West African ICEMR is to analyse epidemiologic, clinical and molecular data on the transmission and human impact of malaria from longitudinal cohort studies (Fig. 1) at sites that differ in the seasonal prevalence of *Plasmodium falciparum* infection and the incidence of disease (uncomplicated malaria) [21]. Based on that rationale, the specific aims of the West African ICEMR are to identify and resolve obstacles to malaria control and its ultimate elimination, i.e., to analyse data outcomes in West Africa — the region of the world with the most intense transmission and the highest incidence of malarial disease and poverty.

In this paper, we describe the development of a DCMS by the West African ICEMR and the lessons learned from that experience, provide practical guidelines and discuss the challenges involved in developing and implementing a DCMS in malaria-endemic countries with severely limited resources. We also discuss the strategies now available to sustain a DCMS by obtaining support from international agencies committed to improving malaria control and achieving its ultimate elimination.

## Methods

**Fig. 2:**
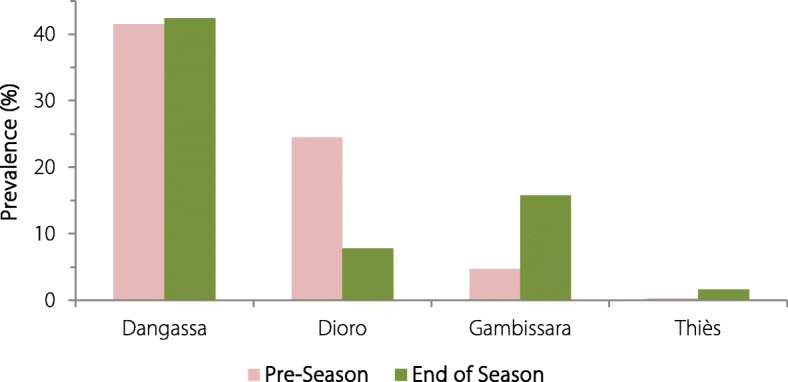
Seasonal changes in the prevalence of *Plasmodium falciparum* infection (based on the frequency of positive thick blood smears). The prevalence of *P. falciparum* infection was high both before and at the end of the season in Dangassa (> 40%). However, it was low (< 2%) before and at the end of malaria season in Madina Fall. Only Gambissara demonstrated the expected pattern, modest low levels of infection (5%) before the malaria season and a substantial increase to 16% at the end of the season. In contrast, the prevalence of infection in Dioro actually decreased between the beginning and end of the malaria season (from 25 to 8%). Pre-season prevalence bars are in red; end of season prevalence bars are in green

**Fig. 3:**
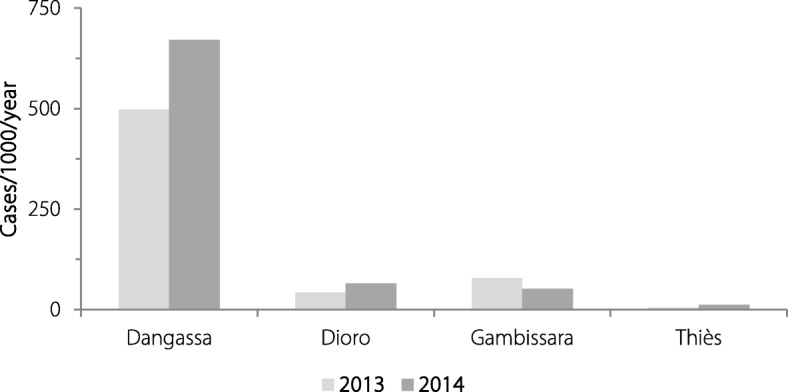
Annual incidence of uncomplicated malaria in a longitudinal cohort. The annual incidence of uncomplicated *P. falciparum* malaria was highest in Dangassa, 10-fold lower in both Dioro and Gambissara and 100-fold lower in Madina Fall. Bars for the incidence of uncomplicated malaria are light gray for 2013 and dark gray for 2014

This study included two rural communities in Mali (Dangassa and Dioro), one in The Gambia (Gambissara) and an urban community in Senegal (Madina Fall in the city of Thiès). Cohort-based household surveys used thick smears to estimate the prevalence of *P. falciparum* infection (active case detection: ACD) from 2012 to 2017 (Fig. 2). Surveys for *P. falciparum* infection were performed at the start and end of the rainy season (in June–August and December–January). In contrast, passive case detection (PCD) was performed every day at each of the community health centres serving the four study sites and provided free diagnosis and treatment with ACTs for cohort participants with uncomplicated malaria throughout the year (Fig. 3) [21]. Each of the four cohorts of ~ 1500 subjects was supplemented annually to replace participants lost to follow-up because they had moved or withdrawn from participation. Likewise, a rolling cohort strategy was used to replace the 20% of children less than 5 years of age who “aged out” of the under five cohort each year. Human landing catches for anopheline biting rates [23] and an enzyme-linked immunosorbent assay (ELISA) for circumsporozoite protein [24] were performed to estimate the intensity of transmission based on the entomologic inoculation rate [25]. This was an observational study to examine the epidemiology of malaria and the impact of the malaria control measures implemented at these study sites by the respective Ministries of Health.

### The DCMS

**Fig. 4:**
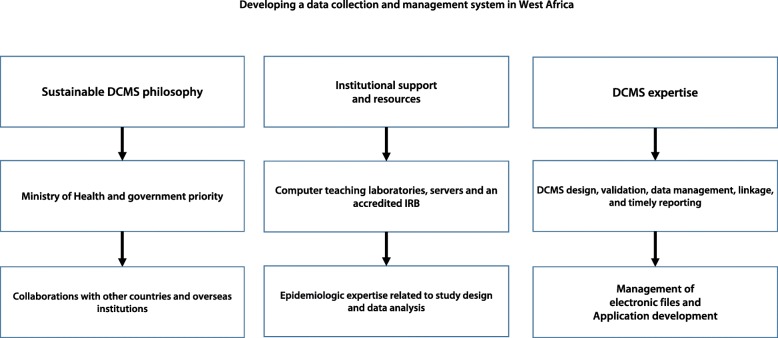
Developing a data collection and management system in West Africa. Development of a regional data collection and management system (DCMS) was based on support from Ministries of Health in the participating countries, WHO, USAID, the President’s Malaria Initiative, the National Institutes of Health and the Centers for Disease Control. Institutional support was provided by the University of Bamako, the University Cheikh Anta Diop in Dakar and the Medical Research Council in Gambia. Computing and epidemiologic expertise were provided by the participating institutions. As a result of the ICEMR workshops, investigators and their DCMS colleagues developed greater expertise in study design, data management and validation, management of electronic files and the development of applications to search the ICEMR database. ICEMR: International Center of Excellence for Malaria Research; IRB: Institutional Review Board; WHO: World Health Organization

**Fig. 5:**
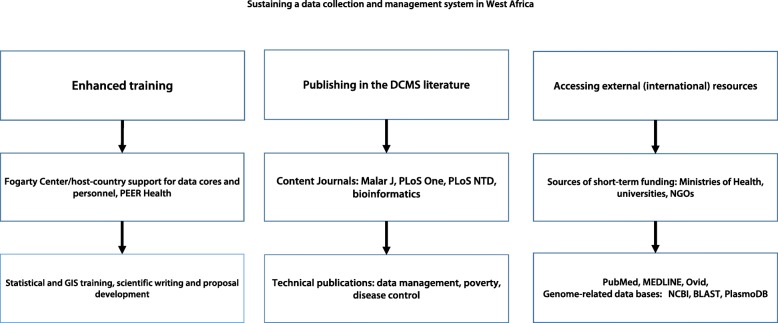
Sustaining a data collection and management system in West Africa. The data collection and management system (DCMS) in West Africa has increased opportunities for training with international (Fogarty, PEER) and host country support, publication (this is the first ICEMR paper on data collection and development of the DCMS) and the ability (opportunity) for West African investigators to access international resources such as Medline and genome-related databases on a regular basis. GIS: Geographic information system; NGO: Non-governmental organisation; NCBI: National Center for Biotechnology Information; BLAST: Basic local alignment search tool; PLoS NTD: PLoS Neglected Tropical Diseases

The individual components of the DCMS developed for these studies (Fig. 4) and the strategies used to sustain the DCMS (Fig. 5) included:

1. Case Report Forms (CRFs, Tables 1 and 2),
2. Data collection personnel (one investigator at each study site with 4–6 additional persons in the field twice yearly for 10 days to perform follow-up surveys of the participants in the longitudinal study cohorts),
3. Centralized Reporting System linked to the *StudyTRAX* database (*ScienceTRAX LLC*, Macon, GA, USA) which was initially based on manual data collection (with double data-entry) and subsequently on electronic data collection,
4. Data Management Personnel included a full-time Data Manager and two data entry clerks at each study site. The Core Leader for the Biostatistics and Data Management Core was responsible for designing and implementing the DCMS (with support from the *StudyTRAX* Coordinator) and for training and supervising the Data Managers.

**Table 1:** Case report forms (CRFs) for the longitudinal cohort study

CRF # and Title	Information obtained	Treatment, Other Data
CRF 1: Screening and enrollment of cohort participants	Village, Age, Physical Exam, Thick Blood Smear, House #	Chronic Illness with Current Medications, Informed Consent
CRF 2: Passive case detection for malarial disease	Census or Study ID #, Date of Visit, Symptoms, Smear	Smear and HRP2-based RDT, Treatment provided for Positives
CRF 3: Microscopy: thick and thin blood smears	Census or Study ID #, Slide Number, Visit Date	Slide Readings and Dates, Microscopist’s Initials
CRF 4: Household and malaria control questionnaire	Census, Household ID #, Questionnaire Responses	Recent IRS, # of Bed Nets, use of Nets and People in the House
CRF 4a: Household questionnaire subform nets	Census, Household ID #, Net Type, Number of ITNs	Net Source, Cost and dipping, Persons sleeping under net(s)
CRF 5: Adult questionnaire	Census, Household ID #, Knowledge about Malaria	Education, Occupation, Malaria Prevention Strategies used
CRF 5a: Adult fever questionnaire subform fever	When did the illness occur? Diagnosis, Treatment	Response to Treatment, Hx of Travel and Bed Net use
CRF 6: Mothers with children < 5 years of age	Census, Household ID #, Hx of Malaria in pregnancy	Treatment of Malaria during previous pregnancies,
CRF 6a: Mothers with children < 5 years of age who have had fever	Mother, Child Census ID #, Diagnosis, Treatment dates	Treatment prescribed, Time to treatment and dosing
CRF 7: Study termination	Reason for Termination: withdrawal, completion, loss	Losses to follow-up: moving, refusals, treatment failure
CRF 8: Chemistry (EDTA) tube tracking for lab testing	Was venous blood obtained for laboratory studies?	Red cell pellet, plasma samples obtained, tested and stored
CRF 9: Twice-yearly follow-up of participants in the longitudinal cohort Study	Census and Study ID #, Hx of malarial illness with a positive smear or RDT,	Diagnosis and treatment: RDT and smear results, clinical response to treatment

*RDT* Rapid diagnostic test

**Table 2:** Case report forms (CRFs) for the entomologic studies

Entomology CRF # and Title	Procedures Performed	Information Obtained
CRF 1: CSP Testing and blood Meal ELISA testing	ELISA testing for CSP antigen and human red cell antigens	Mosquitoes positive for malaria parasites or human red cells
CRF 2: Human landing catches, US CDC light traps	Estimate nightly and monthly anopheline biting rates	Number of infectious bites per person per month (EIR)
CRF 3: Ovarian dissections of captured Anopheline mosquitoes	Dissections performed using stereoscopic microscopes	Distinguish fed, unfed, half-gravid and gravid mosquito vectors
CRF 4: Mosquito species, Molecular forms and resistances	Use PCR to identify *Anopheles* species and *kdr* resistances	Separate *gambiae* from *funestus* and distinguish *kdr* genotypes
CRF 5: Pyrethrum spray catches (PSCs) and analyses	Pyrethroid spraying of houses slept in the night before	PSC estimate of nightly biting rate from # of fed mosquitoes/house

*CSP* Circumsporozoite protein, *ELISA* Enzyme linked immunosorbent assay, *PCR* Polymerase chain reaction

### Development of CRFs and study protocols

The abbreviation CRF [26] is used for both the hard copy (paper) and electronic case report forms (eCRFs) in these studies. The same CRFs were used at all study sites after they had been developed by a panel which included field investigators and data managers from each site, reviewed and approved by the Gambia, Mali, Senegal and Tulane Institutional Review Boards (IRBs) and programmed in *StudyTRAX*. CRFs were prepared and implemented in parallel with project priorities: studies of malaria epidemiology, malaria treatment and entomology (transmission) were designed to define the initial *status quo* and identify potential obstacles to improving malaria control. Paper-based CRFs were used initially because of concerns about the security of electronic data transfers. After those concerns had been resolved by performing a pilot study of electronic data transfers (which showed wire-based transfers could not be detected or hacked using wireless internet access), tablet-based data collection was performed using commercially available (DELL Venue, Round Rock, TX, USA) tablets and hard wire downloads of eCRFs in *Access* via Universal Serial Bus connectors to laptops in the field. These tablets were chosen because of budgetary considerations (rugged laptop computers for use in the field were ≥ USD 4000 apiece) and because the field team had become familiar with the Windows operating system (Microsoft). CRFs were maintained in portable document format (PDF) and editable CRFs were provided only to supervisory personnel. CRF changes, when necessary, were made in March to limit the number of versions circulating and to coincide with the annual review of the Study Protocol for the Longitudinal Cohort Study by the IRBs in April. CRFs were based on closed-ended questions and were anonymous (subjects were identified only by Study ID Number). The data in *StudyTRAX* included links to: 1) country-specific Census ID Numbers and 2) Household ID Numbers for the residence of each subject (which were linked to the entomologic database). Each CRF was also pre-labelled with the subject’s Study ID Number to ensure no data were obtained from persons who had not provided written informed consent for their participation. Although Study ID Numbers were initially hand-written on the CRFs, this process was improved subsequently by using pre-printed labels. Paper (hard-copy) CRFs were completed at the field sites and transported to the four data centres for double (blinded) manual data entry. Global positioning system (GPS) data were collected for each household and retained separately at each study site under lock and key with the signed consent forms required to assign Study ID Numbers.

### Host country data centres

The centres for the four sites provided computer workstations, printers and internet connections in rooms with controlled access. Data centres in Mali and Senegal (for the Dangassa, Dioro and Madina Fall study sites) were located on university campuses (University of the Sciences, Techniques and Technologies of Bamako [University of Bamako] in Bamako and University Cheikh Anta Diop [University of Dakar] in Dakar) and at the Medical Research Council (MRC) Unit in Fajara within their data entry facility for the Gambissara study site [27].

### Database design

*StudyTRAX* provided access to study data on a cloud-based server. The organization and function of *StudyTRAX* are similar to the Research Electronic Data Capture (*REDCap*, Vanderbilt, TN, USA) and *OpenClinica* (OpenClinica LL*C*, Waltham, MA, USA) programs. When funding for these studies became available in 2010, *StudyTRAX* was chosen because *REDCap* was not yet in widespread use [28] and *OpenClinica* did not have off-line data entry [29]. In addition, a template was designed to upload laboratory data to *StudyTRAX* using *Access* queries. The *StudyTRAX* database was equipped with input masks and validation rules for variables available for both on-line and off-line data entry. *StudyTRAX* servers at the African sites, in the cloud, in New Orleans and Austin were used to host and analyse epidemiologic, entomologic, laboratory and clinical data. Copies of database files were uploaded and archived regularly using the *Box* application (*Box, Inc.*, Redwood City, CA, USA). Data linkage was performed using *Access* queries and the *Statistical Analysis System* software (SAS Institute, Cary, NC, USA). Automated monthly reports were developed to monitor double data entry error rates for the Data Core Leader and the members of the Scientific Advisory Group (SAG). These and other functions available through the DCMS were developed incrementally based on teleconferences and on-site training sessions that included the Biostatistics Core Leader, Data Managers, *StudyTRAX* Project Manager, Principal Investigator (PI), Host Country PIs, Project Administrator and other staff.

### Data validation and quality control

All data were validated by performing double-data entry (manual data entry performed by two different [blinded] data-entry clerks) and by using validation rules, range and logic checks and duplicate record checks for all quantitative and categorical variables. The double data entry error reports were used to identify data entry errors, which were then resolved by a supervisor who used the original or electronic copies of original CRFs to identify and select the correct responses. Additional data validation strategies included comparisons between randomly selected CRFs (3–5%) and data in the *StudyTRAX* database. CRF backlogs were calculated by comparing electronic counts of CRFs in the *StudyTRAX* database to the log books at the field sites. Similarly, laboratory results were compared to specimen counts to identify and track backlogs for specimen testing and data entry.

### File sharing, confidentiality and content management

Although the *SharePoint* software (Microsoft) software was considered, the low bandwidths at the field sites (≤250 KBps) made *SharePoint* unsuitable for use at these study sites. As a result, *Dropbox* (Dropbox, Inc., San Francisco, CA, USA) was used in areas with low-bandwidth during the first 3 years of the project because of its simplicity and functionality. During the latter stages of the project, file sharing used *Box* (Box, Redwood City, CA, USA) because of a licensing agreement between *Box* and Tulane University. All metadata, coding guides and CRFs were maintained and archived using *Box*. Confidentiality of the study data was protected in *Box* and *Dropbox* by using Study ID Numbers for all subjects and their data. Records with personally identifiable information such as signed Consent Forms and GPS coordinates for houses were held separately in safes to which only the host-country PI and Data Manager had access and required PI and IRB review and approval for access.

### Data backup

Data backup was based on a four-tiered approach: 1) servers at each host country site (*n* = 4), 2) cloud-based backup on the *StudyTRAX* server (*n* = 1), 3) back-up copies of files on the Tulane University server (*n* = 1) and 4) two additional sets of back-up files on *Dropbox* and *Box* (*n* = 2).

### CRF scanning and archival

CRFs were scanned using Fujitsu ScanSnap iX500 scanners at a rate of 25 pages per minute and a resolution of 600 dots per inch. In practical terms, this resolution was sufficient to read the barcode Study ID labels on more than 95% of CRFs. PDF file names were based on Study ID Numbers to facilitate the searching and organization of the files. Based on this information, the Data Manager in Mali developed an application to manage and query all study PDFs within 10–15 s using *Access*.

### Weekly teleconferences

Teleconferences played a key role in implementing DCMS operations and training. They were held weekly for the Data Managers, Core Leader, *StudyTRAX* Manager, Principal Investigator (PI), Host Country PIs and Project Manager. *GoToMeeting* (LogMeIn, Boston, MA, USA) was used as the platform for these meetings because of its screen-sharing and call-in capabilities. These weekly discussions provided opportunities to validate the database, reconcile data updates, address technical problems and plan data entry and management training. Other teleconference training sessions focused on data management using the SAS University Edition, because it was available free of charge using a Web browser interface.

### On-site training (workshops, Table 3)

The rationale for developing a DCMS in West Africa was to increase the expertise of African Data Managers and Investigators and optimize the function of the DCMS while developing study design and data analysis capabilities on-site for future projects. To achieve those goals, workshops were presented by the Core Leader and his colleagues at the study sites. Workshop attendees included Data Managers, Data Entry Personnel, Project Leaders, Host Country PIs, the Project PI and pre- and post-doctoral trainees. Each study site had a teaching classroom with a projector and internet access, although only one had a computer laboratory. Workshops were delivered in decreasing order of formality, beginning with a formal workshop on Study Design, IRB approvals, Data Entry and Ethical Issues for Investigators, Data Managers and Data Entry Personnel at the start of the ICEMR and concluding with an informal workshop in which the final exercise was the presentation of a draft paper based on recent ICEMR data by each participant (Table 3). Each workshop also included technical components. For participants who did not understand both English and French, host country investigators served as translators. Workshops were presented within one-week (five work days), with 1 day (Friday) reserved for personal assistance and hands-on experience with workshop techniques and software questions. Workshops also provided platforms for Data Managers to present updates on data management and analysis. At the end of each workshop, the attendees received certificates to document their participation.

**Table 3:** Training Workshops held by the West African ICEMR

Workshop theme(s)	Software used	Workshop goals (exercises)	Workshop sites
Data management for ethical Issues, Study Approvals, Case report forms, Data entry, Alternative software packages:	*StudyTRAX* *Generic Packages*	On-line and off-line access to *Study- TRAX* database and data entry *REDCap, OpenClinica*	Dakar, Senegal
Assays for MSP1–42 and AMA-1 Antigens Alternative software packages:	*SoftMax Pro 6* ^a^ *Excel, Access*	Variation in ELISA Titers by Study Site	MRC, The Gambia
Geographic information systems (GIS) Alternative software packages:	*Arc GIS* *Quantum GIS*	Entry and validation of GIS data	Dakar, Senegal
Statistical analysis (Hypothesis Testing) Calculations Data storage	*Stata* *StudyTrax*	Classroom exercises and field data *R, SPSS, University edition of SAS ACCESS*	Bamako, Mali
Paper preparation and submission Graphs and figures	*Microsoft Office*	Tables, graphs, paper drafts	Dakar, Senegal

Training workshops held by the West African International Center of Excellence for Malaria Research

The columns in this table (from left to right) indicate the themes of the workshops, the software packages used for each workshop, the workshops’ goals and the locations (study sites) where the workshops were held

^a^Molecular Devices, LLC – San Jose, CA

#### Data management workshop

This workshop used *StudyTRAX* and *Access* to present topics in data management. The purpose of this training was to guide Data Managers in the use of *StudyTRAX* to generate reports and export data sets for review. In this workshop, participants also learned to use *Access* to combine, manage and query recent data sets.

#### Geographic information systems (GIS) workshop

This workshop covered mapping using both the standard and on-line versions of *ArcGIS* (Esri, Redlands, CA, USA). *ArcGIS* was chosen because of its shapefile formats and the freely available on-line version. Each workshop participant received an introductory *GIS* textbook with an *ArcGIS* license [30] and a limited number of perpetual licenses were available from a previous workshop. Training topics included the use of vector-based spatial data, map design, managing and joining spatial data and map projections. The lack of vector-based imagery for village outlines necessitated the use of web-based imagery such as satellite images for mapping study sites (communities) at both large and small/local scales (e.g., 1:50000 to 1:500).

##### Statistical analysis workshop

This workshop covered hypothesis testing, confidence intervals and statistical modeling using the *Stata* software (StataCorp LLC, College Station, TX, USA). Topics ranged from basic univariate analysis to more advanced techniques such as generalized estimating equations. *Stata* was chosen because it offers a perpetual license (USD500 per license). Data sets included with the documentation and 60-day initial licenses were immensely helpful in providing this training. Participants were provided with an introductory *Stata* textbook [31] and one or more perpetual *Stata* licenses for each site. This workshop also taught the joint use of *Access* and *Stata* because the data management capability and flexibility of *Stata* are limited.

##### Paper writing workshop

This workshop introduced the challenges of scientific writing based on the book *Writing Science* by Schimel (2012) which uses story-telling to teach scientific writing [32]. The content of the workshop focused on papers for high-impact journals and the development of research proposals. Other topics included comparisons of different writing strategies and discussion of the recommended approach: writing from the results in the proposed tables and figures. On the final (fifth) day of the workshop, each trainee presented a draft paper for peer review and discussion.

## Results

Data presented in the Results section below are illustrative, rather than comprehensive. They focus on: 1) results obtained during the initial years of ICEMR support (2012–2014), 2) how those data were used to define the epidemiology of human malaria in West Africa (the initial *status quo*) and 3) how the double data entry software in *StudyTRAX* was used to identify and resolve data entry errors before the data were analysed.

### Functions of the DCMS within the West African ICEMR

Data were collected for 7708 subjects and 918 households to characterize the epidemiology, transmission and human impact of malaria sufficiently to identify obstacles to improved malaria control and its ultimate elimination. Because the initial epidemiologic and entomologic data have been presented elsewhere [14, 15], this paper is about the development of a sustainable DCMS. Therefore, the results section provides an overview of the data gathered, how their quality was assessed, their relation to the laboratory studies performed and the conclusions supported by those data but does not provide detailed comparisons of the biting, sporozoite or entomologic inoculation rates at the different study sites.

### Seasonal changes in the prevalence of *P. falciparum* infection

Based on data from the four field sites (Table 4, Fig. 2), the prevalence of *P. falciparum* infection was highest in Dangassa at both the beginning and the end of the malaria season (41.5%, 42.4%). In contrast, the changes in Gambissara were similar to those at other sites with seasonal transmission: a lower prevalence of infection at the beginning of the season (4.7%) and a higher prevalence at the end of the malaria (rainy) season (15.8%). Although the prevalence of infection had been high in Dioro 10+ years before these studies were performed (when it was similar to the prevalence of infection in Dangassa today), Dioro became part of the Millennium Villages Project (MVP) in 2007 and benefited from MVP support from 2007 to 2014 during the rainy season with ACD, ready availability of ACTs, replacement of worn or torn LLINs and pro-active use of IPTp during pregnancy. With those programmatic changes, the expected increase in the prevalence of infection during the malaria season disappeared and actually became a decrease (from 24.5 to 7.8%, Fig. 2). In contrast, in the urban community of Madina Fall, where malaria is not a major health problem for the residents or health centres, the prevalence of infection was low at the beginning of the season and rose only slightly during the rainy season (from 0.3 to 1.6%) [21].

**Table 4:** Seasonal changes in the prevalence of *Plasmodium falciparum* Infection (based on the frequency of positive thick blood smears)

	Beginning of the malaria season			End of the malaria season		
	Positive	Total	Positive percentage	Positive	Total	Positive percentage
Dangassa	579	1394	41.5	468	1103	42.4
Dioro	365	1487	24.5	95	1218	7.8
Gambissara	65	1397	4.7	194	1225	15.8
Madina Fall	4	1384	0.3	22	1391	1.6
Total	1013	5662	17.9	779	4937	15.8

Seasonal changes in the prevalence of *Plasmodium falciparum* infection (based on the frequency of positive thick blood smears)

Rows indicate the study sites from which blood samples were obtained. Columns indicate the number of samples positive for asexual *P. falciparum* parasites, the number of slides examined and the percent of slides positive by microscopy (columns 2 and 5 were divided by columns 3 and 6 to yield the percent of parasitized subjects in columns 4 and 7, respectively)

### Baseline incidence of uncomplicated malaria (malarial disease)

The data reported above suggest the risk of malarial disease should be greatest in Dangassa, intermediate in Dioro and Gambissara and lowest in Madina Fall. Those expectations are consistent with independent estimates of the annual incidence of malaria based on PCD in 2013–2014, which were 498–671 cases per 1000 persons per year in Dangassa, 43–68 and 52–78 in Dioro and Gambissara, respectively, and 2–12 in Madina Fall (Table 5, Fig. 3) [14, 15]. The differences observed across these four study sites are consistent with the power of a DCMS to identify differences in the prevalence of plasmodial infection and the incidence of malarial disease (i.e., the success vs. failure of malaria control).

**Table 5:** Baseline Incidence of Uncomplicated *P. falciparum* Malaria: West African ICEMR Longitudinal Cohort Study (2013–2014)

	2013			2014		
	Cases by PCD	Cohort × 10^3^	Incidence /10^3^/year	Cases by PCD	Cohort × 10^3^	Incidence /10^3^/year
Dangassa	595	1.194	498.3	722	1.076	671.0
Dioro	55	1.288	42.7	51	0.779	65.5
Gambissara	107	1.370	78.1	69	1.320	52.3
Madina Fall	9	1.615	5.6	18	1.520	11.8
Totals	766	5.467	140.1	860	4.695	183.2

Baseline incidence of uncomplicated *Plasmodium falciparum* malaria

West African International Center of Excellence for Malaria Research longitudinal cohort Study (2013–2014). Columns 2 and 5 indicate the number of persons diagnosed with uncomplicated malaria at each study site during 2013 and 2014 (fever, chills or other symptoms and a positive smear for asexual *P. falciparum* parasites), the number of people participating in the longitudinal cohort study each year (based on unique Study ID Numbers participating in active or passive case detection [ACD or PCD]) and the incidence of uncomplicated malaria as cases per 1000 persons per year (columns 2 and 5 were divided by columns 3 and 6 to yield the estimated incidence of uncomplicated malaria in columns 4 and 7)

*PCD* Passive case detection

### Development of the DCMS

Development of the DCMS benefited from a hypothesis-driven approach and the use of defined variables. Daily interactions on-site between the data managers and data entry personnel facilitated the rapid resolution of discrepancies, reduced the turnover of data entry personnel and permitted the correction of errors in real-time with repeat interviews when needed, thus increasing data quality. In addition, considerable time and effort were spent to develop the individual CRFs (Table 1). Because the development and editing of the CRFs required 1–2 years, this study could have benefitted from the use of previously vetted questionnaires, such as those developed by the Malaria Indicator Survey [33].

### Off-line data entry

Off-line data entry was used initially in Mali because of internet bandwidths ≤250 KBps. However, on-line data entry was feasible by 2014 at all study sites (daytime bandwidths ≥500 KBps; night-time bandwidths ≥1 MBps). Therefore, on-line data entry was used at all four study sites from 2015 to 2017.

### Initial and final (corrected) data entry error rates (Table 6)

The initial subject and variable error rates for double data entry were 7.85% to 44.23% (subject error rates) and 0.02% to 2.12% (variable error rates) across the four study sites. After correction of the errors identified by the Double Data Entry Error Report in *StudyTRAX*, the initial data entry error rates were converted to final data entry error rates of 0% per subject and 0% per variable (Table 6). In practical terms, the rationale for identification and removal of the data entry errors is that the errors removed by this procedure are caused by the human errors that inevitably occur during manual data entry. Please note that this procedure does not affect other study data because it is restricted to questions with double data entry errors which have been confirmed and corrected by a laboratory supervisor.

**Table 6:** Initial results (before) and final results after the correction of data entry errors and error rates

Study Sites	Numbers of subjects	# Subjects with data entry errors before → after	Subject error rates (%) before → after	Data variables entered per study site (#)	Variable Errors per Study Site before → after	Variable error rates (%) before → after
Dangassa	1492	659 → 0	44.17 → 0.00%	1 505 300	23 099 → 0	1.54 → 0.00%
Dioro	1533	678 → 0	44.23 → 0.00%	1 058 853	22 479 → 0	2.12 → 0.00%
Gambissara	1566	123 → 0	7.85 → 0.00%	1 432 503	212 → 0	0.015 → 0.00%
Madina Fall	1868	311 → 0	16.65 → 0.00%	1 475 704	400 → 0	0.027 → 0.00%
Totals	6459	1771 → 0	27.42 → 0.00%	5 472 360	46 190 → 0	0.84% → 0.00%

Final data entry error rates after correcting double data entry errors

Rows indicate the sites from which data were obtained (column 1), the number of subjects participating in the cohort at each study site (column 2), the number of subjects with double data entry errors both before and after correction (left and right sides of column 3), the subject data entry error rates (number of errors divided by the number of subjects) before and after correction (left and right sides of column 4), the number of data points for study variables entered at each study site (column 5), number of data entry errors for study variables per study site before and after correction (left and right sides of column 6) and the mean number of errors (variable error rate) for each variable as a percent (left and right sides of column 7). The subject and variable error rates in columns 4 and 7 were calculated by dividing the number of errors per subject or study variable by the number of subjects or data points for that variable (columns 3 and 6 were divided by columns 2 and 5)

#### StudyTRAX

Advantages of *StudyTRAX* include commands to save data structures, convenient workspaces to manage files, utilities to generate monthly reports and provide remote supervision of server maintenance and software updates. The flexible user permission module in *StudyTRAX* was helpful because it permitted the participation of investigators from multiple countries who had very different roles in the study. Conversely, the limitations of *StudyTRAX* were that it was not open source software and had a dashboard which had been developed for clinical trials rather than epidemiologic (cohort) studies.

### Electronic data collection

Challenges associated with electronic data collection included the large size of the on-screen keyboard, inadvertent tablet damage when working in the field and the limited number of subjects who could be interviewed before it was necessary to download the files from previous interviews. Equipment maintenance for laptops and scanners was also a challenge because of its more limited availability in West Africa.

### Training

Workshops were delivered with progressively more sophisticated learning objectives and decreasing formality. Each workshop included a technology component, which was its focus and presented most of the technical and conceptual challenges. Although internet bandwidth improved substantially during the study, it was never sufficient for groups of trainees to download large applications simultaneously. Therefore, to conduct the workshops, we downloaded single copies of the application files to mass storage devices the day before the workshops began and shared them with participants by downloading copies of those files from the mass storage devices on the first day of the workshop. Because the workshop participants had active copies of *Microsoft Office* on their laptops, we used *Access* to identify linkages that could not be demonstrated in *StudyTRAX*.

## Discussion

In the studies reported here, we developed a DCMS for cohort-based epidemiologic studies of malaria in West Africa. Although this was/is an essential step for the performance of population-based studies, the successful development of a DCMS has additional benefits beyond the performance of individual studies. Those benefits include the development of host country expertise in: 1) the evaluation (analysis) and interpretation of population-based studies and clinical trials and 2) study design and data analysis relevant to the diseases endemic in LMICs.

Please note that the DCMS data and conclusions based on those data were consistent with the other information available about these communities. Gambissara, in the Upper River Region of The Gambia, has more intense transmission than the coastal (lower river) region of The Gambia based on the numbers of persons treated, although comparative population-based (incidence) data had not been available previously. The prevalence of infection in this population increased between the start and end of the malaria season as in most communities with seasonal malaria transmission (Table 4, Fig. 2). Conversely, malaria was uncommon in the peri-urban community of Madina Fall in Senegal, consistent with one small breeding site in the entire community. In contrast, both Dangassa and Dioro were different from most communities with seasonal malaria transmission. Despite the implementation of standard control measures (LLINs, ACTs, IPTp), the prevalence of infection (positive smears) was high throughout the year in Dangassa which had an annual incidence of 500–700 cases of malaria per 1000 persons (Table 5, Fig. 3). Although the data in Dioro had been similar to Dangassa in 2006, in 2007 the Millennium Villages Project began providing intensive malaria control to Dioro and other communities in the Segou Region of Mali. Those interventions (which included ACD and pro-active replacement of worn or torn bed nets) decreased the incidence of disease and the prevalence of infection (positive smears) so markedly in Dioro that the prevalence of infection actually fell during the rainy season (Table 4, Fig. 2).

Because of its roles in study design, data collection and data interpretation, access to a DCMS is essential for public health planning based on rational (evidence-based) priorities. However, as noted above, a DCMS is difficult to support with the short-term, disease-specific funding that is most readily available. In practical terms, this means the support to develop and sustain a DCMS must often be provided from institutional funds — which are typically less available in malaria-endemic LMICs such as Mali, Senegal and The Gambia than in Europe, Asia, Australia, North America or South America. In addition, the cost of software to implement a DCMS and provide training should be considered in relation to its sustainability. For example, the *ArcGIS* and *Stata* software used in the workshops both require licenses, which was a limitation. However, as noted in Table 3, software packages such as *R* (R Foundation for Statistical Computing, Vienna, Austria) and *Quantum GIS* (QGIS, Open Source Geospatial Foundation, Chicago, IL, USA) provide more economical alternatives for statistical analysis and the performance of *GIS* studies in areas such as sub-Saharan Africa.

### Scientific and logistic rationales

In addition to defining public health priorities, an active DCMS allows investigators in LMICs to compete for extramural funding, which encourages the training of host-country investigators focused on unsolved scientific and logistic health problems in their home countries. In our experience, the most important point of leverage to accelerate this process and thus facilitate the long-term retention of highly-trained host country nationals has been to nurture collaborations that involve host-country and international investigators in collaboration with host country Ministries of Health and schools of medicine and public health.

As noted by Kouyaté and Sauerborn in their discussions of the Heidelberg-Nouna institutional affiliation [34, 35], genuinely reciprocal collaborations have the potential to transform the understanding of both partners in the collaboration sufficiently to improve the quality of the health data and may also improve the health of the host-country population.

Because developing, malaria-endemic countries typically have limited resources, the development of regional facilities is the most financially feasible way to proceed initially. One example is the African Centers of Excellence in Bioinformatics Program. This public-private partnership leverages the expertise and facilities of the African Center of Excellence for Bioinformatics in Mali to offer advanced training in bioinformatics to investigators from across Africa [36]. Regional approaches such as this also help to counter the loss of talented investigators (“brain drain”) that can be a limiting factor for capacity building in LMICs [37].

For large-scale international efforts such as the improvement of health across a resource-limited continent, candidate interventions can be (and often are) developed in non-endemic areas (such as the USA, Europe, Australia or Asia). However, once a candidate intervention has been shown to be effective in the laboratory and safe in human subjects, it must be tested for efficacy and safety in human subjects with the disease of interest. In practical terms, this means the efficacy and safety (Phase 2) testing of candidate interventions must be performed in malaria-endemic areas where investigators with expertise in the conduct of clinical trials may be uncommon, facilities to perform clinical trials in compliance with US Food and Drug Administration guidelines may be rare and the networks necessary for multi-site clinical trials have been virtually non-existent. Please note, however, that this situation is changing. Publications are now appearing which describe the training of clinical investigators [38], the conduct of clinical trials [39] and the development of networks to perform multi-site clinical trials in resource-limited areas such as West Africa [40].

The provision of support to investigators from malaria-endemic countries with limited resources and the investigators who developed the candidate interventions is logical and is potentially the most economical way to determine which candidate interventions are efficacious and safe enough to use in disease control programs in malaria-endemic areas to reduce the morbidity and mortality of diseases such as malaria.

## Conclusions

Development of a regional DCMS in West Africa has led to the sharing of study protocols, case definitions, CRFs and reporting guidelines. Error rates have been reduced (data quality improved) by electronic identification of errors on double data entry. In addition, the pooling of data from parallel studies at different sites has improved our understanding of treatment success and encouraged the use of molecular markers to distinguish new infections (which do not indicate treatment failure) from recrudescences with the original parasite genotypes/molecular markers (which do indicate treatment failure).

Because the numbers of health professionals in malaria-endemic areas are currently insufficient, training is essential for the development of a DCMS. Training is also important because it simultaneously provides new opportunities and encourages the long-term retention of highly-skilled host-country investigators.

The expertise available through a DCMS is essential to develop regional centres of excellence. In fact, in the absence of a DCMS or its equivalent, it is difficult to imagine a centre of excellence in the health sciences in any environment. Although this principle is universal, in impoverished, malaria-endemic areas such as West Africa there is an ever-present risk that the immediate short-term need for patient care may interfere with and overwhelm the planning and allocation of the resources necessary for development of DCMSs and the training of host-country investigators necessary for successful institution building in the long-term.

## Definitions

Acceptable Ranges: values expected in normal human subjects such as hemoglobin (Hb) levels of 13–17 g/dl in men and 12–15 g/dl in women and serum sodium levels of 135–145 mM; Bandwidth: available internet capacity expressed as the number of data bits that can be moved per second such as 10 megabits per second (10 MBps) which is 10 million bits of data per second; Data System Metrics: storage capacity (in MB, GB or TB), processing speed (in GHz) and the rates at which data can be downloaded or uploaded (in KB, MB or GB per second) objectively define the function/capacity of a data system; Data Collection and Management System: system into which data can be entered manually or electronically; Gross Domestic Product: total value of all goods and services produced in one country per unit time (year or quarter); INDEPTH Network: global network of research centers performing longitudinal health and demographic studies of populations in LMICs; Input Masks: guidelines to ensure data for each variable are in comparable units (e.g., g/dl for Hb, blood glucose in mg/dl or mM); Outer Limits: limits which should raise questions about the validity of the data, e.g., weights > 100 kg or ages > 90 years in sub-Saharan Africa; Scientific Advisory Group: investigators recommended by an ICEMR and approved by NIH to review ICEMR progress twice or more per year; Validation: review of data outside acceptable ranges or outer limits to ensure those data are valid (this may require the review of individual participant records and specific laboratory or clinical data).

## Additional file

Additional file 1: Multilingual abstract in the five official working languages of the United Nations. (PDF 446 kb)

## Abbreviations

ACD: Active case detection
ACT: Artemisinin combination therapy
CRF: Case report form
DCMS: Data collection and management system
GDP: Gross domestic product
GIS: Geographic information system
GPS: Global positioning system
ICEMR: International Center of Excellence for Malaria Research
IPTp: Intermittent preventive treatment (of malaria) during pregnancy
IRB: Institutional Review Board
LLIN: Long-lasting insecticidal net
LMICs: Low and middle income countries
PCD: Passive case detection
PDF: Portable document format
PI: Principal investigator
WHO: World Health Organization
