# Supplementary material for: Development of a data collection and management system in West Africa: challenges and sustainability
Source: Infect Dis Poverty. 2018 Nov 16;7:125. doi: 10.1186/s40249-018-0494-4 (PMC6292095; doi:10.1186/s40249-018-0494-4)
Supplement: Supplementary file 1 — Multilingual abstract in the five official working languages of the United Nations. (PDF 446 kb) [file 40249_2018_494_MOESM1_ESM.pdf]

Translation of the abstract into the five official working languages of the United Nations

وضع نظام لجمع وإدارة البيانات في غرب أفريقيا: التحديات والاستدامة

جيفري ج. شافير، سيدو و. دومبيا، داودا ندياي، أيوبا ديارا، جولز ف. غوميز، ديفيس نواكانما، إسماعيلة أبو بكر، عبدالله أحمد، منى عفرة، ماري لوكووسكي، كلاريسا فاليم، جيمس س. ويلتي، فرانسيس ج. مآثر، جوزيف كيتنغ ودونالد ج. كروجستاد

مُلَخَّصٌ

خلفية: من الصعب تطوير واستدامة نظام جمع البيانات وإدارته (DCMS) في البلدان الموبوءة بالملاريا بسبب القيود المفروضة على عرض النطاق الترددي للإنترنت وموارد الحاسوب وأعداد الموظفين المدربين. يقوم هذا البحث على فرضية مفادها أن تطوير نظام جمع بيانات وإدارته في غرب أفريقيا كان أحدى النتائج البالغة الأهمية لمراكز الإمتياز الدولية في افريقيا الغربية في مجال أبحاث الملاريا. تتمثل أغراض هذا البحث في إتاحة تلك المعلومات للباحثين الآخرين وتشجيع الربط بين نظم جمع البيانات وإدارتها والبحوث الدولية وأنظمة بيانات ونظم تخزين وزارة الصحة.

الأساليب: وقد قمنا بتصميم وتنفيذ نظام جمع بيانات وإدارتها لربط مواقع الدراسة في مالي والسنغال وغامبيا. يستند هذا النظام إلى نماذج تقرير الحالة للجوانب الوبائية والحشرية والسريرية والمخبرية لعدوى المتصورات ومرض الملاريا لإجراء دراسة أتراب طولية وشملت التدريب الموقعي للباحثين الرئيسيين ومديري البيانات. بناءاً على هذه التجربة، نقترح وضع مبادئ توجيهية لتصميم واستدامة نظم إدارة المعلومات في البيئات محدودة الموارد والموظفين.

النتائج: من عام 2012 إلى عام 2017، أجرينا مسوحات نصف سنوية مكثفة لتشويه اللطاخات للعدوى في المتصورات، ومجموعات البعوض لمعدلات اللدغ الأنوفيلي ومعدلات الحيوانات البوغية والكشف السلبي على مدار السنة لمرض الملاريا في أربع مجموعات طولية مع 7708 فرد و 918 أسرة في السنغال وغامبيا و مالي. وشملت التحديات الرئيسية وضع تعريفات موحدة وتقارير، وتقييم معدلات الخطأ في إدخال البيانات، وعدم القدرة على الوصول إلى الإنترنت وعدم استقرارها، وصيانة البرمجيات والتكنولوجيا. وشملت نقاط القوة ربط مجموعات الحشريات بالدراسات الطولية للأتراب ومراكز البيانات في الموقع ومستودع بيانات المستندة إلى موقع كلاود.

الاستنتاجات: في الوقت الذي يكون فيه البحث على أمراض الفقر في البلدان المنخفضة والمتوسطة الدخل أولوية عالمية، فإن الموارد المتاحة لضمان جمع دقيق للبيانات وأتاحة هذه البيانات إلكترونياً تبقى محدودة للغاية. استناداً إلى تجربتنا، فإننا نقترح وضع نظام إقليمي لجمع البيانات وإدارتها. ويعتبر هذا النهج أكثر اقتصادا من مراكز البيانات المنفصلة ولديه القدرة على تحسين نوعية البيانات من خلال تشجيع مشاركة تعاريف الحالة واستراتيجيات التحقق من صحة البيانات والنهج التحليلي بما في ذلك التحليل الجزيئي لاوجه نجاح العلاج و فشله.

Translated from English version into Arabic by Heba Kandel and Mais Salsa, through

TRANSLATORS WITHOUT BORDERS

在西非（疟疾流行区）开发数据收集和管理系统的挑战及可持续性

Jeffrey G. Shaffer, Seydou O. Doumbia, Daouda Ndiaye, Ayouba Diarra, Jules F. Gomis, Davis Nwakanma, Ismaela Abubakar, Abdullahi Ahmad, Muna Affara, Mary Lukowski, Clarissa Valim, James C. Welty, Frances J. Mather, Joseph Keating and Donald J. Krogstad

摘要

引言：由于互联网带宽、计算机资源和技术人力资源的限制，在疟疾流行国家开发和维持数

据收集和管理系统(DCMS)难度很大。在西非开发 DCMS 是西非国际疟疾研究卓越中心的一个至关重要的成果。本文旨在向其他研究人员提供这类信息，并促进 DCMSs 与国际研究和卫生部数据系统及存储库的联动。
方法：我们设计并开发了一个 DCMS，连接马里、塞内加尔和冈比亚研究地点的数据。基于疟原虫感染和疟疾病例报告的流行病学、昆虫学、临床和实验室方面的数据，该系统进行纵向队列研究，并纳入对主要调查人员和数据管理人员的现场培训。基于这一经验，我们提出了在资源和人员有限的环境中开展 DCMSs 的设计及其可持续发展的指导方针。
结果：2012 年至 2017 年，我们在塞内加尔、冈比亚和马里的 918 户、7708 个体中开展 4 项纵向队列研究，进行厚涂片检测疟原虫感染率、收集蚊媒测定蚊虫叮咬率和孢子虫感染率，全年开展疟疾病例被动检测，全。主要挑战包括制定统一的定义和报告形式，评估数据录入错误率，网络连接的不稳定性和限制，以及软件和技术的维护。该项目的优势在于昆虫数据与纵向队列研究、现场数据中心和基于云的数据存储库的关联。
结论：在低收入和中等收入国家开展对贫困所致疾病的研究是全球重要事宜，然而仍严重缺乏可用资源以确保收集数据和电子数据的准确性。根据我们的经验，我们建议开发一个区域性的 DCMS。这种方法比单个的数据中心更经济，并且有可通过鼓励共享诊断标准、数据验证策略和分析方法(包括治疗成功和失败的分子学分析)来提高数据质量。

Translated from English version into Chinese by Xin-Yu Feng, edited by Jin Chen

CHINESE CENTER FOR DISEASE CONTROL AND PREVENTION
NATIONAL INSTITUTE OF PARASITIC DISEASES

Développement d'un système de collecte et de gestion de données en Afrique de l'Ouest: défis et durabilité

Jeffrey G. Shaffer, O. de Seydou Doumbia, Daouda Ndiaye, Ayouba Diarra, Jules F. Gomis, Davis Nwakanma, Ismaela Abubakar, Adjahi, Muna Affara, Mary Lukowski, Clarissa Valim, James C. Welty, Frances J. Mather, Joseph Keating et Donald J. Krogstad

Résumé
Contexte: Développer et maintenir un système de collecte et de gestion de données (SCGD) est difficile dans les pays où le paludisme est endémique en raison des limitations en ce qui concerne les bandes passantes Internet, les ressources informatiques et le nombre de personnel formé. Cet article part de l'hypothèse que le développement d'un SCGD en Afrique de l'Ouest était un résultat majeur des Centres internationaux d'excellence d'Afrique de l'Ouest pour la recherche sur le paludisme (West African International Centers of Excellence for Malaria Research). Ce document a pour objectif de rendre ces informations disponibles aux autres chercheurs et d'encourager la mise en rapport des SCGD avec les systèmes et recueils de données des recherches internationales et du Ministère de la Santé.
Méthodes: Nous avons conçu et implémenté un SCGD afin de lier les sites d'études du Mali, du Sénégal et de la Gambie. Ce système était basé sur des formulaires d'exposés de cas pour les aspects épidémiologiques, entomologiques, cliniques et de laboratoires d'infection plasmodiale et de paludisme pour une étude de cohorte longitudinale; il comprenait aussi une formation sur place pour les chercheurs principaux et les gestionnaires de données. En nous basant sur cette expérience, nous

proposons des directives pour le développement et le maintien de SCGD dans des environnements à ressources et personnel limités.

Résultats: De 2012 à 2017, nous avons effectué des examens semestriels de goutte épaisse pour l'infection plasmodiale, des collectes de moustiques pour calculer le taux de morsure des anophèles et le taux de sporozoïtes, ainsi qu'une détection passive des cas de paludisme tout au long de l'année dans quatre cohortes longitudinales avec 7708 individus et 918 ménages au Sénégal, en Gambie et au Mali. Les principaux défis comprenaient le développement de définitions et de rapports uniformes, l'évaluation des taux d'erreurs dans la saisie des données, un accès internet instable et limité, et la maintenance des logiciels et des appareils informatiques. Les points forts comprenaient les collectes entomologiques liées aux études de cohorte longitudinale, les centres de données sur place et un référentiel de données basé en nuage.

Conclusions: À l'heure où la recherche sur les maladies liées à la pauvreté dans les pays à faible et moyen revenu est une priorité mondiale, les ressources disponibles pour assurer une collecte de données précise et la disponibilité électronique de ces données restent largement limitée. Sur la base de notre expérience, nous suggérons l'élaboration d'un SCGD régional. Cette approche est plus économique que de séparer les centres de données et elle a le potentiel d'améliorer la qualité des données en encourageant des définitions de cas partagées, des stratégies de validation des données ainsi que des approches analytiques comprenant l'analyse moléculaire des réussites et des échecs du traitement.

Translated from English version into French by Isabelle Redon and Marie Piaget, through

TRANSLATORS
WITHOUT BORDERS

Разработка системы сбора и управления данными в Западной Африке: проблемы и устойчивость

Джеффри Дж. Шаффер, Сейду О. Думбия, Дауда Ндиайе, Аюба Диарра, Джулс Ф. Гомис, Дэвис Нваканма, Исмаэль Абубакар, Абдуллахи Ахмед, Муна Аффара, Мэри Луковски, Кларисса Валим, Джеймс С. Уэлти, Фрэнсис Дж. Мэзер, Джозеф Китинг и Дональд Дж. Крогстад

Аннотация

Общая информация: Разработка и поддержание системы сбора и управления данными (DCMS) является затруднительной в странах, эндемичных по малярии, из-за ограничений в пропускной способности интернета, компьютерных ресурсов и численности квалифицированного персонала. Документ исходит из предпосылки, что развитие DCMS в Западной Африке было критически важным результатом деятельности Западноафриканских международных центров передового опыта в исследовании малярии. Цель настоящей работы — сделать эту информацию доступной для других исследователей и содействовать связи систем DCMS с международными исследовательскими системами управления данными и

хранилищами, а также системами управления данными и хранилищами министерства здравоохранения.
Методы: Мы разработали и внедрили DCMS, чтобы установить связь между исследовательскими центрами в Мали, Сенегале и Гамбии. Эта система основывалась на индивидуальных регистрационных картах по эпидемиологическим, энтомологическим, клиническим и лабораторным аспектам плазмодиальных инфекций, а отакже малярийных заболеваний по продольному когортному исследованию и включала обучение на местном уровне научных руководителей и ответственных за управление данными. Основываясь на данном опыте, мы предлагаем рекомендации по разработке и поддержке DCMS в условиях ограниченных ресурсов и персонала.
Результаты: В период с 2012 по 2017 гг. мы проводили два раза в год исследования толстого мазка на предмет плазмодиальных инфекций, скоплений комаров на предмет уровня анофелиновых укусов и числа спорозоитов, а также круглогодичное пассивное выявление малярийных заболеваний в четырёх продольных когортах, включающих 7708 лиц и 918 домашних хозяйств в Сенегале, Гамбии и Мали. Основные задачи включали разработку единых определений и отчётности, оценки количества ошибок ввода данных, нестабильного и ограниченного доступа к интернету, а также программного обеспечения и технического обслуживания. Сильные стороны включали энтомологический сбор, связанный с продольными когортными исследованиями, местные центры сбора и обработки данных, а также облачное хранилище данных.
Выводы: В то время, как исследование «болезней нищеты» в странах с низким и средним уровнем доходов является глобальным приоритетом, ресурсы, имеющиеся для обеспечения сбора точных данных, а также доступность этих данных в электронном виде по-прежнему сильно ограничены. Основываясь на нашем опыте, мы предлагаем разработку регионального DCMS. Этот подход более экономичен, чем отдельные центры сбора и обработки данных и имеет потенциал для улучшения качества данных путем содействия общим определениям случаев, стратегиям проверки данных и аналитических подходов, включая молекулярный анализ благоприятных и неблагоприятных исходов лечения.

Translated from English version into Russian by Alexander Vareiko and Liudmila Tomanek, through

TRANSLATORS
WITHOUT BORDERS

Desarrollo de un Sistema de Recopilación y Gestión de Datos en África occidental: desafíos y sostenibilidad

Jeffrey G. Shaffer, Seydou O. Doumbia, Daouda Ndiaye, Ayouba Diarra, Jules F. Gomis, Davis Nwakanma, Ismaela Abubakar, Abdullahi Ahmad, Muna Affara, Mary Lukowski, Clarissa Valim, James C. Welty, Frances J. Mather, Joseph Keating and Donald J. Krogstad

Resumen

Antecedentes: Desarrollar y mantener un Sistema de Recopilación y Gestión de Datos (DCMS por sus siglas en inglés) es difícil en países con malaria endémica debido a las limitaciones en el ancho de banda de Internet, los recursos informáticos y la cantidad de personal capacitado. La premisa de este documento es que el desarrollo de un Sistema de Recopilación y Gestión de Datos en África Occidental fue un logro críticamente importante de los Centros de Excelencia Internacional de África Occidental para la Investigación de la Malaria. Los propósitos de este documento son poner esa información a disposición de otros investigadores y alentar la vinculación de los DCMS a los sistemas de datos y repositorios internacionales de investigación y del Ministerio de Salud.

Métodos: Diseñamos e implementamos un DCMS para vincular los sitios de estudio en Mali, Senegal y Gambia. Este sistema se basó en formularios de informes de casos para aspectos epidemiológicos, entomológicos, clínicos y de laboratorio de la infección plasmodial y la enfermedad de la malaria para un estudio longitudinal de cohortes e incluyó capacitación en el sitio para investigadores principales y administradores de datos. Basados en esta experiencia, proponemos pautas para el diseño y la sostenibilidad de los DCMS en entornos con recursos y personal limitados.

Resultados: Entre 2012 y 2017, realizamos estudios bianuales de frotis grueso para infección plasmodial, colecciones de mosquitos para tasas de mordeduras anofelinas y tasas de esporozoitos y detección de casos pasivos durante todo el año para la enfermedad palúdica en cuatro cohortes longitudinales con 7708 individuos y 918 hogares en Senegal, Gambia y Mali. Los principales desafíos incluyeron el desarrollo de definiciones e informes uniformes, la evaluación de las tasas de error de entrada de datos, el acceso a Internet inestable y limitado, y el mantenimiento de software y tecnología. Las fortalezas incluyeron colecciones entomológicas vinculadas a estudios de cohorte longitudinales, centros de datos in situ y un repositorio de datos basado en la nube.

Conclusiones: En un momento en que la investigación sobre las enfermedades que afectan a la pobreza en los países de bajos y medianos ingresos es una prioridad mundial, los recursos disponibles para asegurar la recopilación precisa de datos y la disponibilidad electrónica de esos datos siguen siendo muy limitados. Basados en nuestra experiencia, sugerimos el desarrollo de un Sistema de Recopilación y Gestión de Datos regional. Este enfoque es más económico que los centros de datos independientes y tiene el potencial de mejorar la calidad de los datos mediante el fomento de definiciones compartidas de casos, estrategias de validación de datos y enfoques analíticos, incluido el análisis molecular de los éxitos y fracasos del tratamiento.

Translated from English version into Spanish by Guadalupe Barua and E Passadore, through
